# A feasibility study to investigate post-operative oxygen consumption (POpOC) after colorectal surgery requiring bowel resection

**DOI:** 10.1186/s40814-019-0477-7

**Published:** 2019-07-22

**Authors:** H. E. Taylor, K. Simons, C. Willmott, R. E. R. Smith, D. E. P. Bramley

**Affiliations:** 1 Department Anaesthesia, Pain and Perioperative Medicine, Western Health, 160 Gordon Street, Footscray, Melbourne, VIC 3011 Australia; 2 Centre for Epidemiology and Biostatistics, Melbourne School of Population Health, University of Melbourne, Melbourne, Australia; 3 Western Health Office for Research, Western Health, Melbourne, Australia; 4 Royal Melbourne Hospital, Melbourne, Australia

**Keywords:** Feasibility study, Post-operative, Oxygen consumption, Surgery, Systemic inflammatory response

## Abstract

**Background:**

Oxygen consumption after surgery is increased in response to the tissue trauma sustained intra-operatively and the subsequent systemic inflammatory response that ensues. The cardio-respiratory system must match the tissue oxygen and metabolic requirements; otherwise, peri-operative complications may occur. Existing data is several decades old. The primary objective of this feasibility study was to determine the ease of recruiting participants and collecting relevant data to assess the extent and duration of increased oxygen consumption and post-operative complications after major abdominal surgery in contemporaneous times.

**Methods:**

One hundred patients scheduled for elective colorectal surgery requiring a bowel resection were screened to test specific feasibility criteria relating to ease of recruitment, duration of post-operative stay, ease of data collection, and drop-out rates. A calibrated metabolic cart was used to obtain unblinded pre-operative resting oxygen consumption recordings. The metabolic cart was then used to obtain post-operative oxygen consumption readings on days 1 to 5 as long as the participant remained as an inpatient. At the time of the oxygen consumption reading, a Post-Operative Morbidity Survey score (POMS) was calculated. Feasibility outcomes chosen a priori were that at least one participant would be recruited every 2 weeks from the pre-admission colorectal clinic, at least 10% of potential subjects screened would be enrolled, at least 80% of recruited participants would have a minimum post-operative stay of 2 nights, a minimum of 3 consecutive days of oxygen consumption data would be collected for each subject, at least 8 of 9 POMS score domains would be completed per participant per day and the drop-out rate would be no greater than 10%. We deemed that screening 100 patients would be sufficient to test our feasibility outcomes.

**Results:**

Twelve participants completed the protocol. All pre-specified feasibility criteria were met. No increase in post-operative oxygen consumption was observed in this feasibility cohort.

**Conclusions:**

The protocol and experiences gained from this feasibility study could be used to plan a larger study to better define changes in post-operative oxygen consumption after major abdominal surgery utilizing current surgical techniques.

## Background

Resting oxygen consumption is influenced by several factors including the consumption and digestion of food, environmental temperature, the performance of muscular work, pregnancy, and hormones [1, 2]. Increased post-operative oxygen consumption is driven by a systemic inflammatory response (SIR) to tissue trauma sustained during surgery [3–7]. The primary goal of the cardiorespiratory system is to deliver adequate oxygen to the tissues to meet their metabolic demands [8]. It has been hypothesized that a range of perioperative complications including wound infections, anastomotic leaks, respiratory, and cardiac complications may increase when tissue metabolic demands are unable to be met and tissue hypoxia occurs [9–12]. The use of pre-operative functional capacity assessment to determine the risk of postoperative complications is based on this pathophysiologic rationale [13] although recent evidence has disputed its predictive utility [14]. Earlier studies have followed a limited post-operative period and have not explored the relationship between elevated oxygen consumption and post-operative morbidity. These studies conducted in previous decades may not reflect improvements seen with modern surgical, anaesthetic, and post-operative nursing care or changes in general health demographics [4, 5, 15]. The time course of increased post-operative oxygen consumption, its magnitude, and relationship to post-operative morbidity has not been described in this current context. This study aims to determine the feasibility of recruiting participants and undertake relevant data collection to assess the extent and duration of increased oxygen consumption and post-operative complications after major abdominal surgery.

## Methods

### Participants and study design

This prospective observational feasibility study was approved by Western Health’s low-risk human research ethics committee (LNR/15/WH/69). Written and informed consent was obtained from all participants. Inclusion criteria for this study were age > 18 with the ability to provide informed consent, scheduled for elective colorectal surgery requiring bowel resection with an anticipated post-operative stay of at least two nights. Exclusion criteria included participants, in the view of the treating clinician, were likely to require post-operative inotropes or assisted ventilation, those requiring non-elective surgery, those with known thyroid disorders or requiring thyroid medications (including thyroxine, carbimazole, or propylthiouracil), pregnant or lactating women, those already recruited to another trial and those that received a carbohydrate load drink in the 6 h prior to commencement of surgery. At the time of the study, there was no enhanced recovery program for colorectal patients at our hospital.

Feasibility outcomes chosen a priori were that at least one participant would be recruited every 2 weeks from the colorectal pre-admission clinic, at least 10% of potential subjects screened would be enrolled, at least 80% of recruited participants would have a minimum post-operative stay of 2 nights, a minimum of 3 consecutive days of oxygen consumption data would be collected for each subject, at least 8 of 9 POMS score domains would be completed per participant per day, and the drop-out rate would be no greater than 10%.

The colorectal preadmission clinic includes patients presenting for a number of surgeries, including many that do not require bowel resection, such as peri-anal procedures, hernia repairs, endoscopy, and reversal procedures; hence, the modest recruitment target of at least 10% of all patients attending this clinic. Accounting for the heterogeneous surgical cohort, we determined that screening 100 patients would be sufficient to test our pragmatic feasibility outcomes and achieve a convenience sample of more than 10 patients.

### Measurements

A calibrated metabolic cart (Medgraphics Ultima series, model 790705-305) with Breezesuite software (version 6.4.1.53 SPG) was taken to the participant location for all study measurements. Oxygen consumption was obtained by having the participant breathe through a mouthpiece (Hans Rudolph Vbite, part number 602078) and disposable pneumotach (MGC diagnostics, part number 758100-004) with their nose occluded using a nose-clip. Data was collected shortly after each participant was admitted but prior to being taken to theatre with the subject fasting and resting in a semi-recumbent position. Measurements were conducted over 5 min in total to allow for averaging of results. A baseline recording was performed on each participant’s day of surgery, prior to them being taken to theatre.

Subsequent recordings were performed in the morning on the surgical wards with the participant fasting for at least 6 h and in bed. Recordings were repeated in the same manner on post-operative mornings 1–5, as long as the participant remained an inpatient. At the time of the oxygen consumption recording, a Post-Operative Morbidity Survey (POMS) score was calculated. POMS scores are a validated measure of post-operative morbidity after major abdominal surgery [16]. The investigators were unblinded to all recordings. When a participant was using supplemental oxygen post-operatively, the oxygen was disconnected for 5 min prior to the oxygen consumption recording commencing. Peripheral oxygen saturations were monitored via a portable pulse oximeter (Datex Ohmeda TuffSat, part number 6051-0000-148) concurrently with the aim to cease the metabolic cart measurement if the SpO_2_ fell below 90% while breathing room air. Ambient and subject temperatures were recorded at the time of oxygen consumption measurement.

### Statistical methods

Demographic data were presented as number, range, and proportion as appropriate. Feasibility outcomes were described as having been met or not. Oxygen consumption data was presented as mean and standard deviation and POMS scores presented as median and range. For each participant, we summarised the change in oxygen consumption over baseline into an ‘area under the curve’ by using the trapezium rule. In this summary measure, we included only the days with complete follow-up for all participants (days one to three) to avoid selection bias. We used a one-sample *t* test to test for a change in oxygen consumption in this post-operative period and repeated the analysis using day one post-operation minus baseline. Analyses were performed using the R statistical package version 3.4.2

## Results

**Fig. 1:**
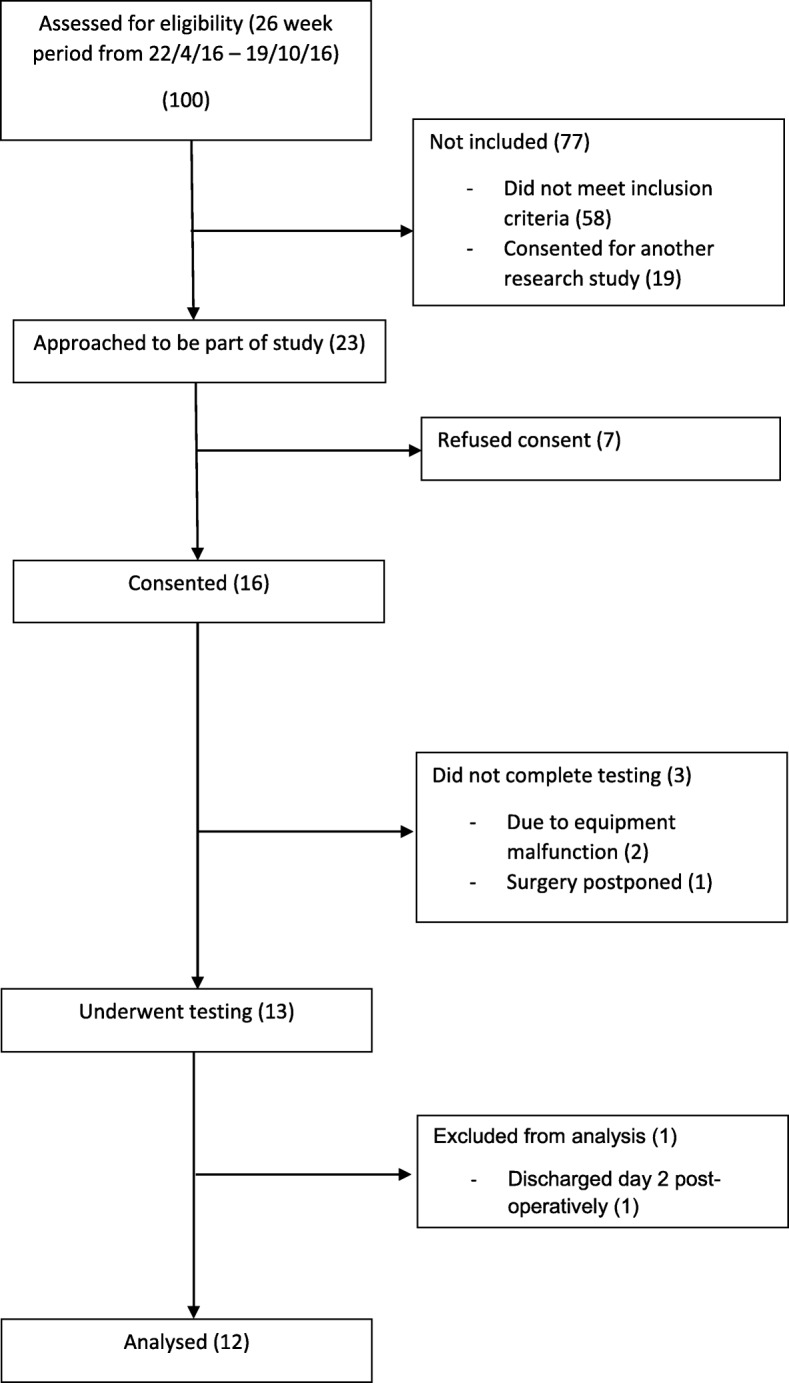
Participant recruitment and protocol completion

A total of 100 consecutive potential participants attending the colorectal pre-admission clinic between April and October 2016 were assessed for eligibility. Fifty eight patients did not meet eligibility criteria, the majority of which were scheduled for procedures that did not require a bowel resection 23 were approached and 16 were enrolled giving a recruitment rate of 70% (95% CI 47–87) to achieve 12 complete records. The pre-specified rate of recruitment was achieved, and relevant oxygen consumption measures, POMS scores, and demographic data were able to be recorded for all participants. Three participants were unable to complete the planned measures after recruitment; two due to calibration and metabolic cart malfunction issues, and one had their surgery postponed. A total of 13 participants underwent testing. One participant was excluded from analysis due to being discharged day 2 post-operatively, leaving a total of 12 participants who underwent analysis (see Fig. 1). One participant declined to continue on their fifth post-operative day, citing fatigue in the context of a post-operative ileus. All other participants had complete datasets. Subject characteristics are shown in Table 1. All patients received a general anaesthetic without supplementation with a neuraxial analgesic technique.

**Table 1:** A summary of participant characteristics and surgical information. Values are mean (SD), median [range], or number (proportion)

	*n = 12*
Age, years	61.7 (9.21)
Sex	
Male	7 (58%)
Female	5 (42%)
BMI*	30 (6.13)
Pre-operative hemoglobin, g/L	125 (21.9)
ASA**physical status score	
1	2 (16.7%)
2	8 (66.7%)
3	2 (16.7%)
Cancer surgery	
Yes	10 (83%)
No	2 (17%)
Surgical technique	
Laparoscopic assisted	10 (83%)
Open (converted)	2 (17%)
Surgical procedure	
Right hemi-colectomy	6 (50%)
Anterior resection	3 (25%)
Ultra-low anterior resection	1 (8%)
Hartmann’s procedure	1 (8%)
Sub-total colectomy	1 (8%)
Duration of procedure in min (skin incision to closure)	190 (69.7)
Length of stay in days	5.41 (2.96)
POMS*** score day 5	0 [0-2]
Ambient temperature at time of measurement in °C	23.6 (1.11)
Participant temperature at the time of measurement in °C	36.7 (0.38)
Time between completion of surgery and time of first post-operative oxygen consumption reading in hours	16.5 (2.34)

*BMI body mass index

**ASA American Society Anaesthesiologists

***POMS Post-Operative Morbidity Survey Score

**Fig. 2:**
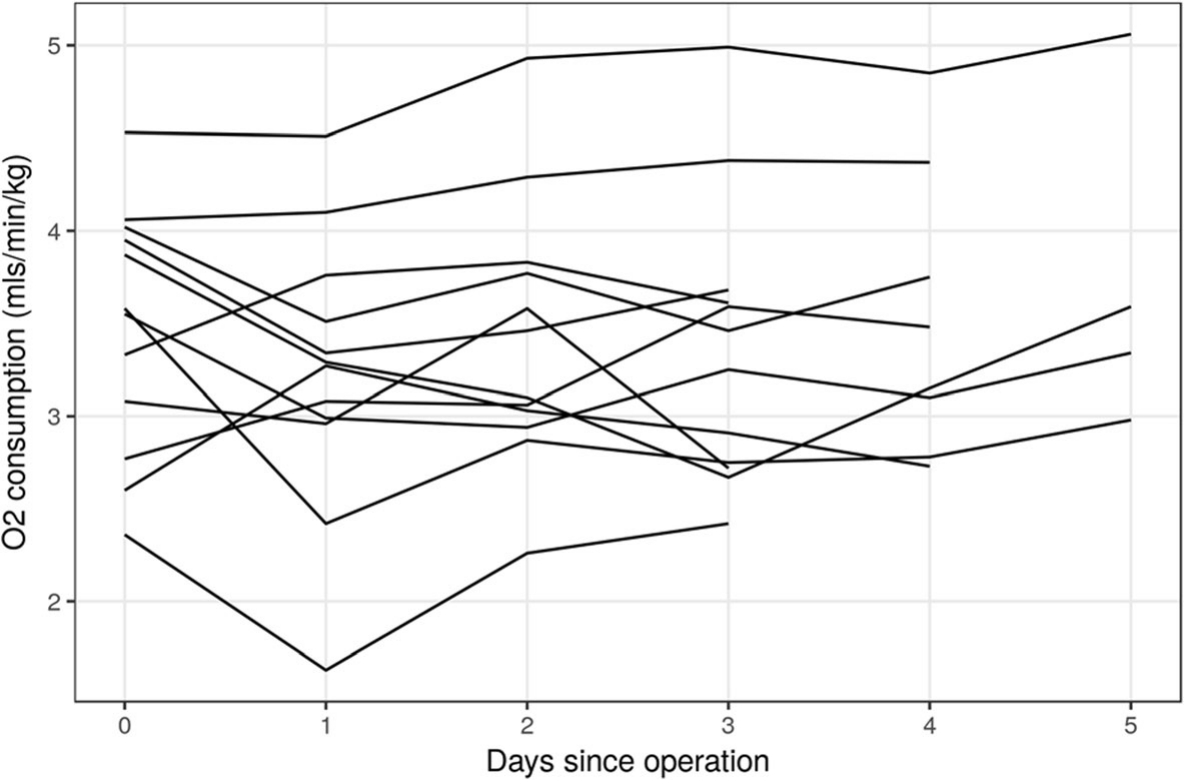
A summary of all participants’ oxygen consumption versus time

Individual participant oxygen consumption is presented in Fig. 2. Table 2 shows the average consumption per day as well as the average (pairwise) difference between day 1 and pre-op, and the average area under the curve (AUC). There was no clinically apparent increase observed in post-operative oxygen consumption out to day 5. Noting that this study was not designed or powered to test this hypothesis, analysis revealed a non-significant decrease in oxygen consumption (AUC − 0.34 ml/min/kg [− 1.13; 0.45] *p* = 0.37—first day − 0.24 ml/min/kg [− 0.59; 0.11] *p* = 0.16).

**Table 2:** Oxygen consumption and Post-Operative Morbidity Survey scores vs time. Values are mean (SD) or median [range]

	Pre-operative	Day 1	Day 2	Day 3	Day 4	Day 5	AUC*	Delta**
O_2_ consumption (ml/min)	296 (67.2)	272 (57.2)	290 (56.2)	284 (55.6)	287 (65.6)	302 (57.9)	-37.6 (1.2)	-24.6 (50.4)
O_2_ consumption (ml/min/kg)	3.48 (0.63)	3.24 (0.72)	3.43 (0.68)	3.37 (0.72)	3.53 (0.71)	3.74 (0.79)	-0.34 (1.2)	-0.24 (0.5)
Median POMS***		2 [1-3]	1 [0-3]	1 [0-3]	1 [0-3]	0 [0-2]		

*AUC area under the curve restricted to the first 3 days of observation (days*ml/min respectively days*ml/min/kg)

**Delta difference between day 1 and pre-operative measurement

***POMS Post-Operative Morbidity Survey score

## Discussion

This study was able to meet all the pre-specified feasibility criteria, and this study protocol could be used to guide a larger study. There was no apparent increase in post-operative oxygen consumption in our cohort, although the small sample size, lack of pre-specified significance levels, and appropriate power calculations preclude any conclusions being drawn from this observation [17].

We attempted to control for confounders by measuring and excluding factors that may have had an influence on oxygen consumption, such as ambient temperature and hormonal influences.

It is possible that our data collection protocol could miss an increase in post-operative oxygen consumption if it occurred prior to the first oxygen reading with a subsequent return to baseline. Increasing the observation frequency would be logistically challenging given the requirement for participants to be fasted at the time of measurement and availability of trained staff to collect data. Post-operative oxygen consumption on day 1 was measured in the morning, regardless of whether the patient had their surgery in the morning or afternoon on the previous day, meaning the time between the end of surgery and oxygen recording was variable. However, previous data has shown an increase in post-operative oxygen consumption of up to 20–44% for several days post-operatively, reaching a peak at 24 h post-operatively [4, 5, 15]. Our sample of size 12 had more than 80% power to detect a difference of such magnitude with a two-sided *t* test, whereas smaller effect sizes require much larger sample size, e.g., a 10% increase in post-operative oxygen consumption would require a sample size of 22 patients and for 5% as many as 79 patients are needed. While these power calculations require assumptions, such as the absence of sub-groups of patients who respond differently, the lack of observable increase in oxygen consumption seen in our study suggests that perhaps the SIR after modern surgery is not as pronounced as previous studies have demonstrated. This hypothesis is supported by recent work showing that increases in post-operative cortisol levels, another marker of post-operative stress, are not as high as previous studies had shown [18]. Future studies could potentially try to get an earlier first post-operative reading of oxygen consumption to see if an increase is occurring within the first 12–24 h. Alternatively, future studies could be designed using an equivalence design to anticipate no change in post-operative oxygen consumption.

Limitations of this study include the technical difficulties that were encountered by the researchers using the metabolic cart. This was particularly in relation to getting adequate calibration prior to testing; however, the researchers wanted to ensure the precision of the results. Another difficulty encountered was having a member of the research team available to collect data early on a weekend. We did not examine all factors impacting oxygen delivery apart from hemoglobin and oxygen saturations. However, it is likely that the oxygen consumption seen in our cohort was supply independent and therefore unlikely to impact our results [19, 20], in contrast to the relationship between oxygen delivery and consumption in septic patients [19].

This study was a feasibility study, and while we did not see an increase in oxygen consumption our results should be interpreted with caution given the small sample size. The oxygen use measured in this study was a representation of global consumption and cannot delineate between regional or organ differences. The pattern of perioperative oxygen consumption and its relationship with patient morbidity is not well defined.

## Conclusions

This feasibility study has demonstrated that our protocol for measurement and data collection was able to be successfully implemented at our institution in patients undergoing major intra-abdominal surgery. The experience and data provided could be used to explore this relationship in a larger study.

## Data Availability

The datasets used and/or analyzed during the current study are available from the corresponding author on reasonable request.

## Abbreviations

ASA: American Society Anaesthesiologists
AUC: Area under the curve
BMI: Body mass index
IQR: Inter-quartile range
POMS: Post-Operative Morbidity Survey score
SD: Standard deviation
SIR: Systemic inflammatory response
